# Role of diagnostic stewardship in reducing healthcare-facility–onset *Clostridioides difficile* infections

**DOI:** 10.1017/ash.2022.305

**Published:** 2023-03-16

**Authors:** Anita B. Shallal, Medha Cherabuddi, Lance Podsiad, Christopher Gortat, Clare Shanahan, Tarlisha Holsey, Linoj Samuel, George Alangaden, Geehan Suleyman

**Affiliations:** 1 Division of Infectious Disease, Henry Ford Health, Detroit, Michigan; 2 Wayne State University School of Medicine, Detroit, Michigan; 3 Department of Internal Medicine, Henry Ford Health, Detroit, Michigan; 4 Information Technology, Henry Ford Health, Detroit, Michigan; 5 Infection Prevention and Control, Henry Ford Health, Detroit, Michigan; 6 Clinical Microbiology, Henry Ford Health, Detroit, Michigan

## Abstract

We describe the implementation of an electronic medical record “hard stop” to decrease inappropriate *Clostridioides difficile* testing across a 5-hospital health system, effectively reducing the rates of healthcare-facility–onset *C. difficile* infection. This novel approach included expert consultation with medical director of infection prevention and control for test-order override.

*Clostridioides difficile* infection (CDI) is the most common cause of healthcare-associated infectious diarrhea in the United States.^1^ For National Healthcare Safety Network (NHSN) reporting, CDI is defined as positive *C. difficile*diagnostic test (toxin or molecular assay) on unformed stool samples, irrespective of the cause of diarrhea.^2,3^ Thus, healthcare facility-onset (HCFO) CDI reporting is a laboratory-identified (LabID) event.^3^ Rates of HCFO-CDI considered a quality metric by the Centers for Medicare and Medicaid Services (CMS) directly affect hospital reimbursement.^2^ The annual CDI-attributable cost in the United States exceeds $6 billion, and a diagnosis of CDI increases the cost of hospitalization by 54%, estimating $34,157 per case.^4^

Rates of asymptomatic colonization with *C. difficile* can be as high as 15% in healthy adults, and risk factors include previous CDI, prior hospitalization, and use of immunosuppressants and steroids.^5^ Nucleic acid amplification test (NAAT) for *C. difficile* has a sensitivity >90%^6^; however, this test does not distinguish infection from colonization. Diarrhea that is not related to *C. difficile* is common in hospitalized patients, especially in the setting of promotility agents.^7^ Inappropriate testing for *C. difficile* in patients with non-CDI–related diarrhea is estimated to occur up to 40% of the time^7^ and may lead to inaccurate diagnosis of CDI in colonized patients, unnecessary treatment, prolonged hospitalization, and increased healthcare costs.

In 2019, ∼45% of all HCFO-CDI occurred in patients receiving promotility agents in our institution. In this study, we assessed the effectiveness of an electronic medical record (EMR) “hard stop” in reducing inappropriate CDI testing and its impact on HCFO-CDI rates.

## Methods

In this before-and-after quasi-experimental retrospective study, we compared *C. difficile* test order rates per 1,000 patient days, CDI rate per 1,000 patient days, and standardized infection ratio (SIR) between the preintervention period (January 2018 to December 2019) and the intervention period (April 2020 to March 2022) in a 5-hospital healthcare system in southeastern Michigan.

A multistep algorithm with enzyme immunoassay (EIA) for toxin A/B and glutamate dehydrogenase antigen followed by NAAT for discordant EIA results is utilized in our institution.

**Fig. 1.**
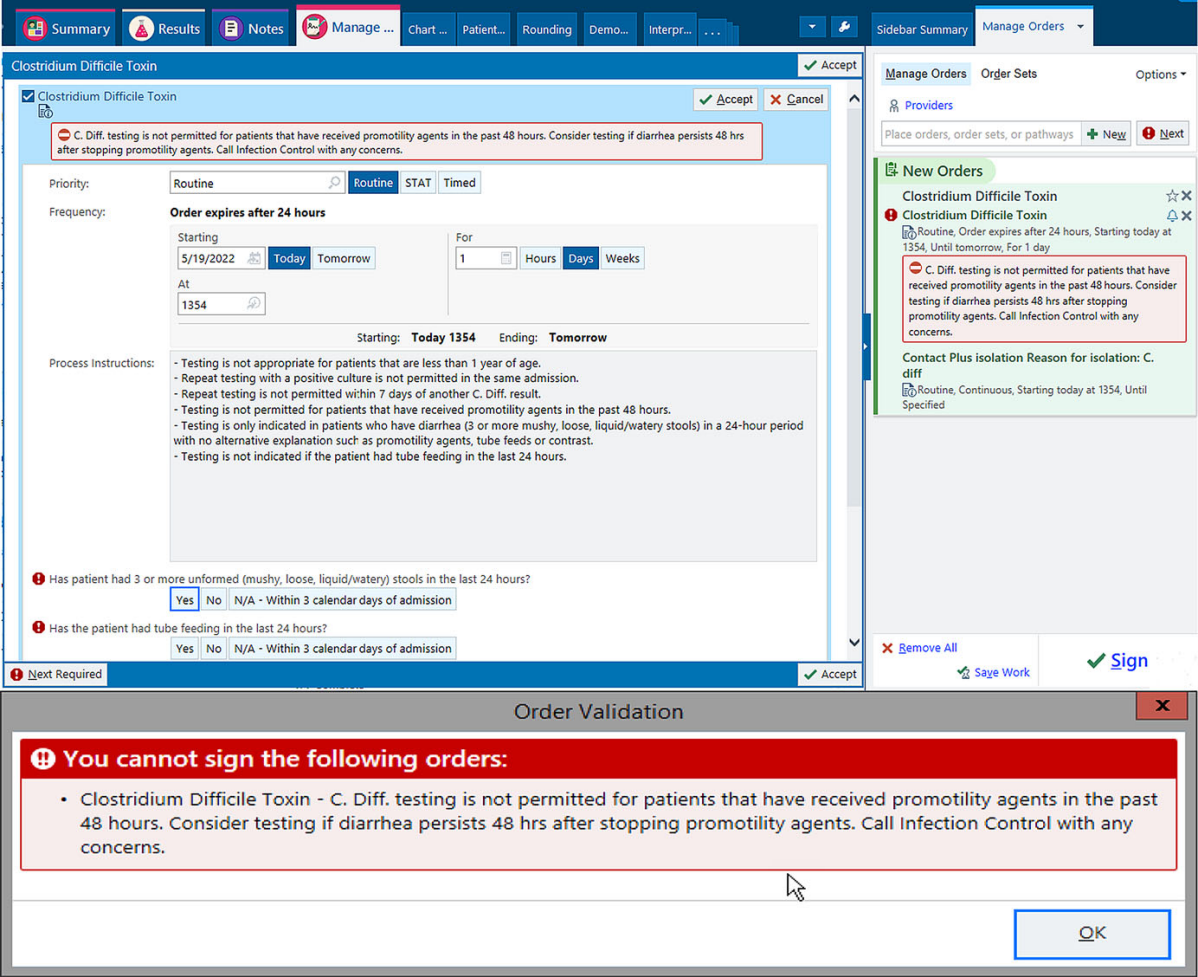
Screen of the *Clostridium difficile* testing order and subsequent hard stop encountered upon order validation in the electronic health record.

In January 2020, systemwide education regarding the electronic *C. difficile* test-order hard stop in Epic software (Epic, Verona, WI) was provided to frontline staff. The hard stop went live in February 2020 and was automated to appear >3 days after admission upon signing the order in the following settings: receipt of promotility agents within 48 hours; patients aged <1 year; repeated testing within 7 days for negative results or during the same admission for positive results. See Supplementary Table 1 for laxative groups; magnesium oxide was also included. Reasons for the hard stop and instructions on whom to contact if testing was desired were provided (Fig. 1). The medical director of infection prevention and control, or designee, could override the hard stop after reviewing the case upon provider request. After discontinuing the promotility agents for ≥48 hours, providers were able to place an order if diarrhea persisted. For patients on tube feeding, consultation with dietician to address diarrhea was recommended prior to testing. All orders were cancelled after 24 hours if a specimen was not collected. Override requests were retrospectively reviewed to determine the positivity rate among them.

## Results

**Fig. 2.**
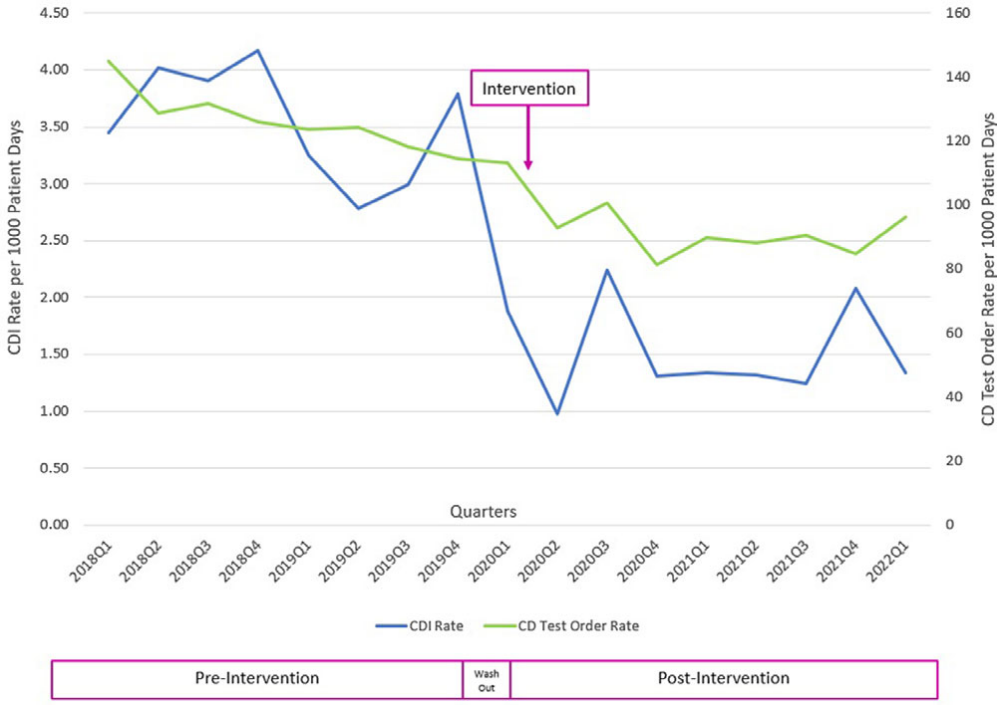
*Clostridium difficile* infection (CDI) and *C. difficile* (CD) test order rates before and after the intervention.

The CDI rates per 1,000 patient days were 3.54 in the preintervention period and 1.48 in the postintervention period, a 58% reduction (Fig. 2). The test order rate per 1,000 patient days was 126.5 in the preintervention period and 90.6 in the postintervention period, a 28% reduction (Fig. 2). The SIR decreased from 0.521 in the preintervention period to 0.347 in the postintervention period, a 33% reduction (95% confidence interval, 0.56–0.79; *P* < .001). Of the 289 overrides, 41 tests (14%) were cancelled due to lack of specimen collection and 248 tests (86%) were performed. Of those performed, 26 (11%) were positive (20 toxin tests and 6 NAATs). All patients who tested positive were treated for CDI. The most common reasons for overrides were diarrhea in critically ill patients with sepsis and cirrhotic patients in whom laxatives could not be discontinued. Community-onset (CO) CDI rates decreased from 1.05 in the preintervention period to 0.88 in the postintervention period, a 16% reduction.

## Discussion

Utilization of an electronic hard stop was effective in reducing unnecessary *C. difficile* testing, resulting in lower HCFO-CDI rates with no reported adverse events associated with delayed testing. Decision making for CDI testing is challenging and requires astute clinical judgement given the lack of specificity for causes of diarrhea in inpatients.^7^ In this population, CDI accounts for just 10%–20% of cases of diarrhea, whereas medications, including promotility agents, antimicrobials, and tube feeding account for the overwhelming majority.^8^ Due to highly sensitive NAAT that cannot distinguish colonization from infection, restricting testing to patients with no alternative causes for diarrhea is essential in preventing inappropriate diagnosis of CDI and overtreatment.

Diagnostic stewardship is an effective strategy for reducing inappropriate *C. difficile* testing in inpatient settings.^8–10^ Soft stops, such as clinical decision support algorithms and best practice alerts (BPAs), are commonly used to mitigate unnecessary testing, especially in the setting of promotility agents. However, hard stops that actively block orders are underutilized despite being more effective.^9,10^ Solanky et al^8^ implemented measures, including a test-ordering algorithm outlining patient criteria for *C. difficile* testing incorporated into the EMR, which reduced inappropriate testing. However, clinicians were able to order testing even if criteria were not met.^8^ In another study, a BPA was triggered in various settings, including laxative administration in the preceding 48 hours. Overriding the BPA activated a hard stop in which testing could not proceed without microbiology laboratory approval. The laboratory approved all orders upon request and provided a passcode for new order entry.^10^

Unlike other studies, our testing hard stop was triggered by objective measures captured in the EMR. Our intervention was also novel because it included a consultation with the medical director of infection prevention upon provider request if testing was denied. This expert review of individual cases provided for prompt feedback, education and shared decision making. Providers were encouraged to address confounding factors, including discontinuation of promotility agents, and explore alternative causes.

After the intervention, we also evaluated CO-CDI rates to ensure that there was no increase due to possible delayed testing. CO-CDI rates decreased, and no patients were readmitted with CDI due to delayed testing. In addition to being safe and effective, the intervention had the additional benefit of quality improvement and cost savings for the health system, with an estimated net savings of nearly $7 million.^4^

Given the retrospective quasi-experimental nature of this study, these results are subject to inherent limitations. Additionally, the number of hard stops that were fired is unknown, making it difficult to gauge the true number of reduced *C. difficile* test orders. For test orders for which an override was approved, the reasons for override were not collected. Prior studies have noted that the most frequent reason for an inappropriate CD test was reporting of diarrhea by a patient or a nurse.^7^ This information could inform future quality-improvement initiatives.

We observed reductions in *C. difficile* testing, HCFO-CDI rates, and SIR after implementation of an electronic hard stop with optional expert review. Incorporating this strategy into best practices, such as provider education, handwashing, and contact isolation, can significantly reduce HCFO-CDI rates.

## Supplementary material

For supplementary material accompanying this paper visit http://doi.org/10.1017/ash.2022.305. click here to view supplementary material

## References

[1] Ragusa R , Giorgianni G , Lupo L , et al. Healthcare-associated Clostridium difficile infection: role of correct hand hygiene in cross-infection control. J Prev Med Hyg 2018;59:E145–E152. 30083622 PMC6069405

[2] Zaver HB , Moktan VP , Harper EP , et al. Reduction in health care facility-onset Clostridioides difficile infection: a quality improvement initiative. Mayo Clin Proc Innov Qual Outcomes 2021;5:1066–1074. 34820598 10.1016/j.mayocpiqo.2021.09.004 PMC8599925

[3] National Healthcare Safety Network. Multidrug-resistant organism and *Clostridioides difficile* infection. Centers for Disease Control and Prevention website. https://www.cdc.gov/nhsn/psc/cdiff/index.html. Published 2022. Accessed January 10, 2022.

[4] Zhang S , Palazuelos-Munoz S , Balsells EM , Nair H , Chit A , Kyaw MH. Cost of hospital management of Clostridium difficile infection in United States—a meta-analysis and modelling study. BMC Infect Dis 2016;16:447. 27562241 10.1186/s12879-016-1786-6 PMC5000548

[5] Crobach MJT , Vernon JJ , Loo VG , et al. Understanding Clostridium difficile Colonization. Clin Microbiol Rev 2018;31:e00021–17. 10.1128/CMR.00021-17 PMC5967689 29540433

[6] Deshpande A , Pasupuleti V , Rolston DD , et al. Diagnostic accuracy of real-time polymerase chain reaction in detection of Clostridium difficile in the stool samples of patients with suspected Clostridium difficile infection: a meta-analysis. Clin Infect Dis 2011;53(7):e81–e90. 21890762 10.1093/cid/cir505

[7] Kara A , Tahir M , Snyderman W , Brinkman A , Fadel W , Dbeibo L. Why do clinicians order inappropriate Clostridium difficile testing? An exploratory study. Am J Infect Control 2019;47:285–289. 30392996 10.1016/j.ajic.2018.08.019

[8] Solanky D , Juang DK , Johns ST , Drobish IC , Mehta SR , Kumaraswamy M. Using diagnostic stewardship to reduce rates, healthcare expenditures and accurately identify cases of healthcare facility-onset Clostridioides difficile infection. Infect Control Hosp Epidemiol 2021;42:51–56. 32943129 10.1017/ice.2020.375 PMC9215221

[9] Sullivan KV , Gallagher JC , Leekha S , et al. Use of diagnostic and antimicrobial stewardship practices to improve Clostridioides difficile testing among SHEA Research Network hospitals. Infect Control Hosp Epidemiol 2022;43:930–934. 34376271 10.1017/ice.2021.133

[10] Mizusawa M , Small BA , Hsu YJ , et al. Prescriber behavior in Clostridioides difficile testing: a 3-hospital diagnostic stewardship intervention. Clin Infect Dis 2019;69:2019–2021. 31125399 10.1093/cid/ciz295

